# Surgical Management of Ipsilateral Breast Cancer Recurrence After Conservative Mastectomy and Prepectoral Breast Reconstruction: Exploring the Role of Wide Local Excision

**DOI:** 10.3390/cancers17172881

**Published:** 2025-09-02

**Authors:** Lorenzo Scardina, Eleonora Petrazzuolo, Cristina Accetta, Beatrice Carnassale, Sabatino D’Archi, Alba Di Leone, Annasilvia Di Pumpo, Enrico Di Guglielmo, Flavia De Lauretis, Antonio Franco, Federica Gagliardi, Stefano Magno, Francesca Moschella, Maria Natale, Chiara Rianna, Alejandro Martin Sanchez, Marta Silenzi, Gianluca Franceschini

**Affiliations:** Breast Unit, Department of Women, Children and Public Health Sciences, Fondazione Policlinico Universitario Agostino Gemelli IRCCS, 00168 Rome, Italy; eleonora.petrazzuolo@policlinicogemelli.it (E.P.); cristina.accetta@policlinicogemelli.it (C.A.); beatrice.carnassale@guest.policlinicogemelli.it (B.C.); sabatino.darchi@policlinicogemelli.it (S.D.); alba.dileone@policlinicogemelli.it (A.D.L.); annasilvia.dipumpo@policlinicogemelli.it (A.D.P.); enrico.diguglielmo@policlinicogemelli.it (E.D.G.); flavia.delauretis@guest.policlinicogemelli.it (F.D.L.); antonio.franco@guest.policlinicogemelli.it (A.F.); federica.gagliardi@guest.policlinicogemelli.it (F.G.); stefano.magno@policlinicogemelli.it (S.M.); francesca.moschella@policlinicogemelli.it (F.M.); maria.natale@policlinicogemelli.it (M.N.); chiara.rianna@policlinicogemelli.it (C.R.); martin.sanchez@policlinicogemelli.it (A.M.S.); marta.silenzi@guest.policlinicogemelli.it (M.S.); gianluca.franceschini@policlinicogemelli.it (G.F.)

**Keywords:** breast cancer, local recurrence, prepectoral breast reconstruction, wide local excision, implant breast reconstruction

## Abstract

**Background:** Conservative mastectomy with prepectoral breast reconstruction is becoming increasingly widespread and validated in recent years. Today, while aesthetic advantages and improvement in quality-of-life outcomes are widely acknowledged, oncological safety remains subject of debate. There is limited evidence on residual breast tissue after conservative mastectomy, and it still represents an unknown risk for local recurrence. The recent spread of this surgical technique precludes a standardized surgical approach in case of local recurrence of ipsilateral breast cancer, and the lack of evidence in the literature complicates the decision-making process. The objective of this study is to describe the surgical treatment of local relapses for breast cancer patients following conservative mastectomy and prepectoral implant-based reconstruction. **Methods:** Between January 2018 and May 2024 at a single institution, 648 consecutive patients underwent conservative mastectomy and prepectoral reconstruction as their primary treatment. We identified 12 patients with T1-2 breast cancer who subsequently had histologically confirmed ipsilateral breast cancer recurrence and a local wide excision or radical mastectomy were performed. Each clinical case was discussed in a multidisciplinary meeting to define the most appropriate surgical treatment. At time of diagnosis of recurrence, patients with lymph node metastasis or systemic involvement were excluded from the study. **Results:** Among 648 consecutive patients who underwent conservative mastectomy, 12 with histologically confirmed ipsilateral breast cancer recurrence were included. The mean interval to recurrence was 43 months (range 10–76 months) from the primary operation. Recurrence sites were as follows: upper outer quadrant in four patients (33.4%), upper inner quadrant in three (25.0%), lower inner quadrant in two (16.6%), lower outer quadrant in one (8.4%), and central quadrant with nipple involvement in two (16.6%). Of the 12 patients, 9 (75%) underwent wide local excision, including 2 who also received partial capsulectomy, while 3 (25%) required radical mastectomy with implant removal. Adjuvant radiation therapy was administered to 6 patients (50%)—5/6 (83.3%) in the excision group and 1/6 (16.7%) in the mastectomy group. No significant differences were observed in distant disease–free survival or overall survival between the two groups. **Conclusions:** Currently, surgical treatment of ipsilateral breast tumor recurrence following conservative mastectomy and prepectoral breast reconstruction is not reported in the literature, and this study represents the first instance where wide local excision is described. The management of ipsilateral recurrence should be discussed in multidisciplinary meetings and could be performed safely in selected cases, sparing the prosthesis and avoiding radical mastectomy.

## Simple Summary

This retrospective study examines the surgical management of ipsilateral breast cancer recurrence following conservative mastectomy with prepectoral reconstruction, a scenario for which evidence and standardized guidelines remain scarce. Radical mastectomy has traditionally been regarded as the standard approach, yet our findings suggest that wide local excision may represent a feasible and oncologically safe alternative in selected cases. This strategy not only preserves the implant in most patients but also reduces surgical invasiveness, with no apparent compromise in cancer-related outcomes. Multidisciplinary evaluation proved essential for treatment planning, ensuring an individualized balance between oncologic safety and preservation of reconstructive results. By providing early data in this underexplored field, our study may contribute to refining future clinical decision-making and optimizing care for patients experiencing local recurrence.

## 1. Introduction

Conservative mastectomy, often referred to as nipple- or skin-sparing mastectomy, has gained increasing popularity in recent years, particularly when combined with prepectoral implant-based breast reconstruction (IBBR) [1,2,3,4]. This approach offers significant aesthetic and psychological benefits by preserving the skin envelope and avoiding disruption of the pectoralis major muscle, thus enhancing patient satisfaction and quality of life [5,6,7,8]. As a result, it has become a standard surgical option for selected patients with early-stage breast cancer [9,10,11,12]. Despite its advantages, conservative mastectomy raises important concerns regarding oncological safety [13,14,15]. Residual breast tissue may persist along the skin flaps or around the nipple-areolar complex, posing a theoretical risk for local recurrence [16]. While local recurrence rates after nipple- or skin-sparing mastectomy are generally low, the lack of standardized surveillance protocols and surgical guidelines for recurrence management in reconstructed breasts presents a clinical challenge [17]. Candidates for prepectoral reconstruction must be carefully evaluated, and the breast surgeon is expected to perform the conservative mastectomy following the correct anatomical plane, thus preserving skin flaps with adequate subcutaneous fat thickness while ensuring complete removal of glandular tissue [18,19,20,21]. In a previous study, we have already demonstrated that conservative mastectomy followed by prepectoral implant-based reconstruction is oncologically safe, yielding outcomes comparable to those of traditional submuscular techniques [22,23]. In particular, the optimal surgical approach to ipsilateral breast cancer recurrence (IBCR) following conservative mastectomy and prepectoral implant reconstruction remains undefined [24]. Historically, radical mastectomy with implant removal has been considered the standard of care [25,26]. However, in cases of limited and localized recurrence, wide local excision (WLE) may represent a less invasive alternative, potentially allowing for preservation of the implant and surrounding tissues without compromising oncologic safety [27]. To date, there is a paucity of data specifically addressing the surgical management of IBCR in patients with prepectoral breast implants. The current literature lacks prospective comparisons, and available evidence is largely limited to small retrospective series [28,29,30].

This study aims to contribute to filling this gap by analyzing the clinical characteristics, surgical treatment, and oncologic outcomes of patients with IBCR after conservative mastectomy and prepectoral breast reconstruction. Our goal is to assess the feasibility and safety of WLE in this unique clinical context and to inform future treatment guidelines for managing recurrence in reconstructed breasts.

## 2. Materials and Methods

This retrospective study was conducted at Fondazione Policlinico Universitario Agostino Gemelli IRCCS in Rome, Italy. Medical records of patients who underwent conservative mastectomy with prepectoral IBBR between January 2018 and May 2024 were reviewed. Among these, we identified patients who subsequently developed histologically confirmed IBCR. This study received the approval of the Lazio-3 Ethics Committee on 27 February 2025 (ID number: 7424), and each patient gave informed consent for operation and clinical data acquisition.

### 2.1. Inclusion and Exclusion Criteria

Eligible patients were women aged >18 years with a prior diagnosis of invasive breast carcinoma treated by nipple-sparing mastectomy (NSM) or skin-sparing mastectomy (SSM), followed by immediate prepectoral IBBR and a confirmed ipsilateral local recurrence. Patients with distant metastatic disease at the time of recurrence were excluded.

### 2.2. Patient and Data Collection

Patients were categorized into two groups based on surgical management of the recurrence: wide local excision (WLE) and radical mastectomy (RM). Collected data included demographic characteristics, tumor biology (histological subtype, grade, receptor status), time to recurrence, location of recurrence, imaging findings, type of surgical treatment, adjuvant therapies, and oncologic outcomes including disease-free survival (DFS) and overall survival (OS).

### 2.3. Management Algorithm

**Figure 1:**
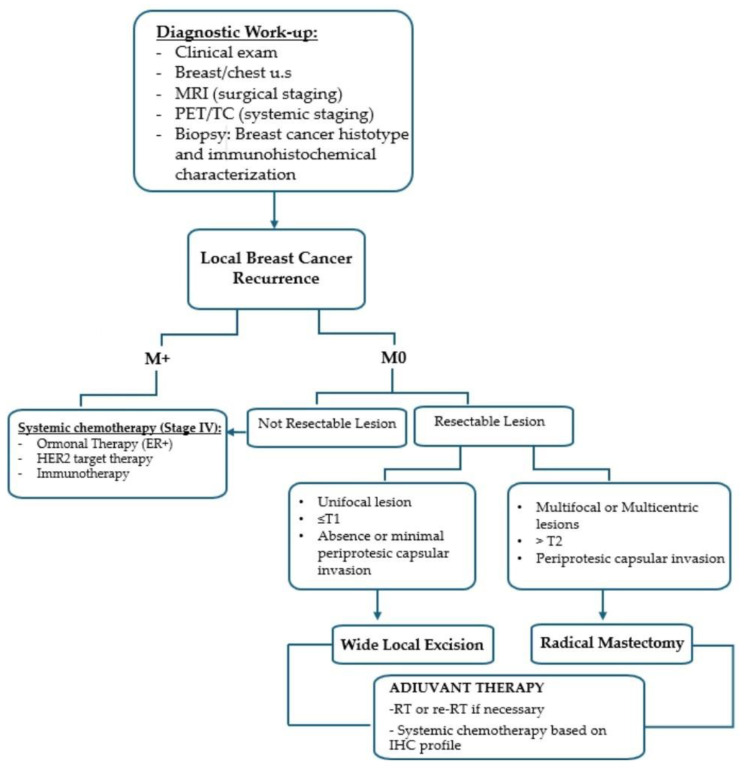
Algorithm for multidisciplinary management of patients with locoregional recurrence after conservative mastectomy.

Figure 1 outlines the clinical algorithm used for the diagnosis and surgical management of IBCR. All patients underwent a comprehensive diagnostic work-up, including clinical breast examination, breast and chest wall ultrasound, and contrast-enhanced breast MRI for surgical planning. Whole-body PET/CT was performed to exclude distant metastases. Diagnosis was confirmed by image-guided core needle biopsy with full histopathological and immunohistochemical analysis. Patients were initially categorized as metastatic (M1) or non-metastatic (M0) based on PET/CT findings. M1 patients were referred for systemic therapy, including endocrine therapy, HER2-targeted therapy, or immunotherapy according to molecular subtype. M0 patients were evaluated for surgical resectability. The choice of surgical procedure in M0 patients was based on recurrence characteristics:

• WLE was considered in cases of unifocal recurrence (≤T1) without or with minimal periprosthetic capsule invasion.
• RM was indicated for multifocal/multicentric disease, tumor size > T2, or clear involvement of the periprosthetic capsule.

### 2.4. Surgical Treatment

**Figure 2:**
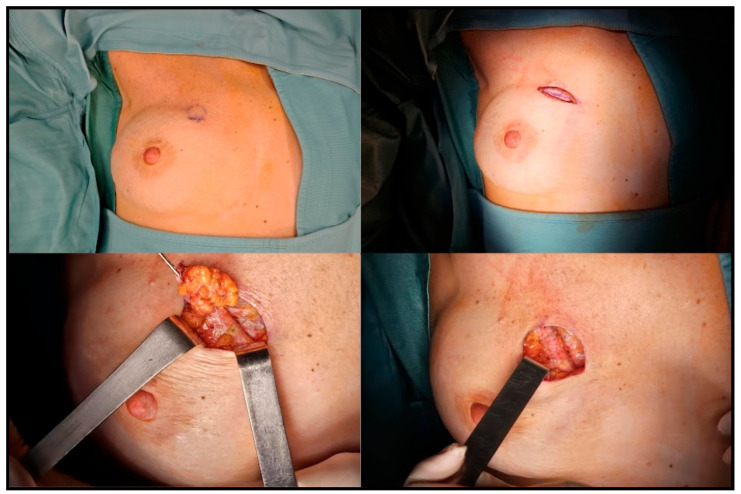
Sequential steps of the wide local excision procedure following conservative mastectomy and prepectoral prosthetic reconstruction in a 50-year-old patient with ipsilateral breast cancer recurrence in the upper-inner quadrant.

**Figure 3:**
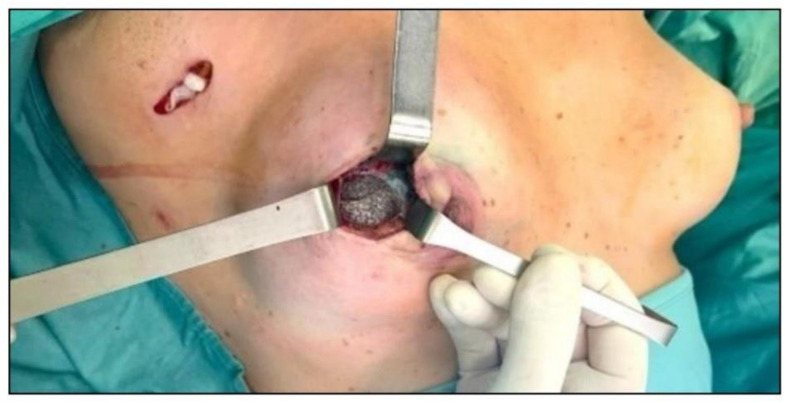
In this case, wide local excision involved not only removal of the recurrent lesion but also partial excision of the periprosthetic capsule.

Two types of surgery were performed based on preoperative staging and imaging:

• Wide local excision (WLE) consisted of en bloc resection of the tumor with clear margins, including an overlying skin ellipse (Figure 2) and, if necessary, partial capsulectomy (Figure 3).
• Radical mastectomy (RM) involved removal of the skin overlying the recurrence, the entire implant, and the surrounding capsule.

WLE was considered for unifocal (≤T1) recurrences and also when there was focal, localized skin involvement directly over the lesion. RM was indicated in the presence of diffuse skin involvement (including nipple–areolar complex), or tumor size > T2, or whenever margin negativity would otherwise be compromised.

The extent of capsular involvement primarily guided implant management: focal capsular contact was addressed with partial capsulectomy within WLE and implant preservation, whereas substantial capsular infiltration precluded safe preservation and prompted implant removal, with conversion to RM if overall disease extent warranted it.

MRI was mandatory in all patients to assess tumor extent, evaluate the relationship with the implant capsule, and determine resectability [31,32]. WLE candidates underwent preoperative lesion localization using either skin marking or Magseed^®^ placement [33]. All patients received perioperative antibiotic prophylaxis according to institutional protocol.

### 2.5. Adjuvant Therapy

Postoperative treatment was personalized based on tumor biology, recurrence characteristics, and previous treatments. Adjuvant radiotherapy or re-irradiation was considered when indicated, and systemic therapy was guided by immunohistochemical profile and clinical risk assessment [34,35]. This individualized algorithm-based approach aimed to balance oncologic safety with the potential to preserve reconstructive outcomes in carefully selected patients.

## 3. Results

Among 648 consecutive patients who underwent conservative mastectomy with immediate prepectoral IBBR, 12 (1.85%) developed IBCR. The mean age at recurrence was 47.3 years, compared to 43.3 years at the time of the primary tumor. The median time to recurrence was 42 months (range: 10–76 months) (Table 1).

### 3.1. Pathological Characteristics

At recurrence, most tumors were classified as pT1 (10 patients, 83.3%), with one case each of pT2 (8.3%) and pT3 (8.3%). No cases of in situ disease were observed at recurrence. In contrast, initial tumors had a broader distribution: pTis in two patients (16.6%), pT0 in two (16.6%), pT1 in six (50%), and pT2 in two (16.6%). The predominant histological subtype at recurrence was invasive ductal carcinoma, accounting for 83.3% of cases. Cases of pure ductal carcinoma in situ (DCIS) and other subtypes were infrequent and similarly distributed across both time points. Grade III tumors were observed in 83.3% of patients both at initial diagnosis and at recurrence. Positive nodal status at recurrence was found in two patients (16.6%). Estrogen receptor (ER) status was evenly distributed, with six patients (50%) being ER-positive and six (50%) being ER-negative. HER2 overexpression was identified in four patients (33.4%), and one patient (8.3%) had triple-negative disease. Multifocality at recurrence was rare (one patient, 8.3%) compared to four patients (33.4%) at initial diagnosis. Notably, six patients (50%) experienced recurrence in the same quadrant as the original tumor.

### 3.2. Surgical Technique

Of the 12 patients, 9 (75%) underwent WLE including two who required partial capsulectomy. The remaining three patients (25%) underwent RM with implant removal. Axillary lymph node dissection was performed in three patients (25%), while nine patients (75%) required no axillary procedure. Two patients (16.6%) had received neoadjuvant chemotherapy prior to surgery for recurrence. Regarding implant outcomes, the original implant was preserved in nine patients (75%), removed in two (16.6%), and replaced in one patient (8.4%). Capsulectomy was performed in six patients (50%), while no capsule surgery was performed in the remaining six. Importantly, no postoperative surgical complications, including infection or implant loss, were reported in patients treated with WLE, allowing for full implant preservation in these cases.

### 3.3. Adjuvant Treatment and Oncological Outcomes

Adjuvant therapies following surgery included chemotherapy (5 patients, 41.6%), radiotherapy (6 patients, 50%), hormonal therapy (6 patients, 50%), and immunotherapy (2 patients, 16.6%) based on individual tumor biology. After a median follow-up of 28 months after post-recurrence treatment, all patients were alive and no distant recurrences were observed. There were no statistically significant differences in DFS or OS between the WLE and RM groups.

## 4. Discussion

This study offers one of the earliest in-depth evaluations of surgical strategies for managing IBCR following conservative mastectomy with prepectoral IBBR. The findings suggest that in carefully selected cases, WLE may be a viable alternative to radical mastectomy, preserving the reconstructive integrity while achieving comparable oncologic outcomes. The optimal management of IBCR after conservative mastectomy requires a multidisciplinary approach [36]. Unfortunately, no prospective trials are currently available in the literature to provide high-level evidence for establishing the best treatment strategy. In the review conducted by Buchholtz et al. [37], several prognostic factors were identified that may influence patient outcomes in the context of local recurrence. These include whether the recurrence represents a true local relapse or a new primary tumor—typically inferred from changes in molecular or histopathological profiles, involvement of a different quadrant compared to the primary tumor, or alterations in the gene expression profile. Other relevant prognostic factors include the resectability of the lesion, eligibility for adjuvant radiotherapy, and the possibility of receiving systemic therapies [38,39,40]. In the study by Wu et al. [41], the prognostic significance of loco-regional recurrence (LRR) following nipple-sparing mastectomy and IBBR was thoroughly explored in a large cohort. Their findings revealed a 5-year disease-free survival rate of 73.7% following LRR, indicating that local recurrence does not necessarily equate to poor long-term outcomes. Interestingly, the 5-year post-recurrence DFS varied substantially based on the site of recurrence: patients with nipple–areola complex recurrence exhibited the most favorable prognosis, with a DFS of 89.1%, compared to 73% in those with skin/subcutaneous or chest wall recurrence, and only 59.4% in patients with regional recurrence. These differences likely reflect not only biological heterogeneity but also the invasiveness and clinical detectability of each recurrence subtype. The authors suggest that nipple recurrences, which were more frequently non-invasive, may represent earlier or less aggressive relapses, supporting the importance of recurrence classification in tailoring salvage strategies and estimating prognosis. These data reinforce the idea that selected patients with localized, favorable recurrence patterns may benefit from conservative surgical approaches without compromising oncologic safety. However, it is important to emphasize that all these considerations were drawn from a cohort of patients who underwent conventional mastectomy with submuscular reconstruction, which differs substantially from the population included in our study. The anatomical site of recurrence, particularly central quadrant or nipple involvement [42], plays a critical role in surgical decision-making. Yamaguchi et al. [43] emphasized that nipple recurrences have distinct biological behaviors and may warrant a tailored surgical approach due to their association with more extensive disease [44]. The classification and consistent reporting of recurrence location and characteristics are essential for comparability across studies. The Maastricht Delphi consensus [45] highlighted the need for standardized definitions of local, regional, and distant recurrence, which is particularly relevant when assessing recurrence after conservative mastectomy and IBBR. De La Cruz et al. [46] conducted a systematic review confirming that nipple recurrence rates after conservative mastectomy remain low, and they do not significantly compromise overall survival, suggesting that conservative strategies may be justified in selected cases.

In our experience, only two patients presented with recurrence involving the nipple and were treated with complete excision of the nipple and the underlying tissue. Furthermore, molecular subtype plays a crucial role in predicting recurrence patterns. According to Golijanin et al. [47], patients with triple-negative or HER2-positive subtypes are at higher risk for local recurrence after conservative mastectomy, which could influence the choice of surgical approach at recurrence. In our cohort, four patients were HER2-positive (33.4%) and only one was triple-negative (8.3%). In contrast to previously published data, we did not observe a higher incidence of local recurrence among these subtypes. It is important to note, however, that patients with these tumor profiles were often eligible for advanced systemic treatments, including immunotherapy and targeted biological therapies, which may have been prioritized over surgical treatment [48,49]. Moreover, the inherently aggressive nature of HER2-positive and triple-negative breast cancers may have contributed to a greater likelihood of regional or distant recurrence rather than isolated local recurrence, thereby precluding surgical management in these cases. Joo et al. [50], in their systematic review, found that most local recurrences after reconstruction are manageable, and recurrence patterns do not differ substantially between subpectoral and prepectoral reconstructions. This supports the feasibility of WLE even in the context of a prepectoral implant. Moreover, Siponen et al. [51] demonstrated that with modern multidisciplinary treatment, local recurrence rates remain low after mastectomy, and outcomes after local recurrence are generally favorable, especially when managed conservatively. Goel et al. [52] and Wu et al. [53] both underscore that surgical resection remains the cornerstone of locoregional recurrence management, with WLE being an acceptable option in appropriately selected cases, especially when combined with systemic therapy and radiation.

Notably, axillary management was not addressed in this study, as we believe this topic warrants a separate, dedicated analysis. Our focus was specifically on the surgical approach to IBCR involving the breast, following conservative mastectomy and prepectoral IBBR. Axillary surgery, particularly in the setting of recurrence, should be evaluated independently, taking into account the significant changes in axillary management over the past decade—particularly the widespread adoption of de-escalation strategies—which continue to evolve even in the context of recurrent disease [54,55,56].

Furthermore, it should be noted that all patients who underwent WLE experienced no surgical complications, and implant preservation was achieved in all cases. Notably, in three patients, the procedure included partial resection of the periprosthetic capsule without compromising implant integrity. These findings suggest that, in carefully selected cases, prepectoral IBBR allows for WLE with partial capsular excision while maintaining prosthesis preservation. Although the median follow-up period was relatively short, early oncologic outcomes appeared comparable between the two groups.

This single-center retrospective study includes a very small cohort and a median follow-up of just over two years, which limits broader applicability and may introduce selection bias. Accordingly, the findings should be considered exploratory and hypothesis-generating. As noted, a prospective, multicenter investigation with standardized protocols and longer follow-up is needed to substantiate these preliminary observations

This study demonstrated that, in carefully selected cases of IBCR occurring in patients who had previously undergone conservative mastectomy followed by prepectoral IBBR, WLE may be considered a valid treatment option following thorough multidisciplinary evaluation. This approach allows for implant preservation and contributes to improved quality of life.

It is important to emphasize that this type of management is applicable specifically to patients with prepectoral IBBR, in whom the pectoralis major muscle is not interposed between the skin flap and the implant. Despite the absence of muscular coverage, the integrity of the prosthesis can still be maintained. The variability in recurrence patterns and treatment responses observed in our study highlights the need for a more structured approach to guide surgical decision-making in cases of ipsilateral breast cancer recurrence after conservative mastectomy and prepectoral reconstruction. While our algorithm offers a practical, clinically driven framework for treatment selection, it remains rule-based and retrospective. A future step toward personalized, data-driven care would involve the development of a predictive model integrating multiple clinical, radiological, and pathological variables. Such a model could estimate the likelihood of successful wide local excision versus the necessity for radical mastectomy helping clinicians balance oncological safety with reconstructive preservation. Candidate predictors may include tumor size and location, recurrence timing, MRI-detected capsule involvement, multifocality, histologic subtype and molecular markers (e.g., ER, HER2 status).

This approach could ultimately improve surgical planning, reduce overtreatment, and support shared decision-making, especially in younger patients who value breast reconstruction outcomes. Incorporating patient-reported outcomes in future models would further enhance their relevance in clinical practice.

## 5. Conclusions

In this retrospective, single-center study, WLE was feasible in carefully selected cases of IBCR after conservative mastectomy with prepectoral IBBR, permitting implant preservation.

A key consideration is that the prepectoral setting, characterized by the absence of muscle interposition between the skin flap and the implant, does not preclude adequate resection when careful multidisciplinary assessment is performed. These results are consistent with emerging literature supporting the role of WLE in specific recurrence patterns and reinforce the importance of individualized surgical planning in the context of contemporary reconstructive techniques.

While these data are promising, larger prospective studies are warranted to validate these outcomes and to better define the criteria for patient selection in this evolving clinical scenario.

## Figures and Tables

**Table 1:** Clinicopathological features of the patient cohort according to the type of surgical intervention.

	Overall	WLE (Wild Local Excision)	RM (Re-Mastectomy)
	12	9 (75%)	3 (25%)
**Tumor Classification**			
Tis	1 (8.3%)	1 (11.1%)	1 (33.3%)
T1	9 (75%)	8 (88.9%)	1 (33.3%)
T2	1 (8.3%)	0 (0%)	1 (33.3%)
T3	1 (8.3%)	0 (0%)	0 (0%)
**Histologic Subtype**			
IDC	10 (83.3%)	7 (77.78%)	2 (66.7%)
CDIS	1 (8.3%)	1(11.1%)	0 (0%)
Other	1 (8.3%)	1 (11.1%)	1 (33.3%)
**ER status**			
Positive	6 (50%)	6 (66.7%)	1 (33.3%)
Negative	6 (50%)	3 (33.3%)	2 (66.7%)
**HER2 status**			
Positive	4 (33.4%)	3 (33.3%)	1 (33.3%)
Negative	8 (66.6%)	6 (66.7%)	2 (66.7%)
**Triple Negative**	2 (16.6%)	1 (11.1%)	1 (33.3%)
**Multifocality**			
Yes	1 (8.3%)	3 (33.3%)	1 (33.3%)
No	11 (81.7%)	6 (66.7%)	2 (66.7%)
**Axillary Surgery**			
Yes	3 (25%)	3 (33.3%)	1 (33.3%)
No	9 (75%)	6 (66,7%)	2 (66.7%)
**Systemic Therapy**			
Yes	2 (16.6%)	1 (11.1%)	1 (33.3%)
No	10 (83.4%)	8 (88.9%)	2 (66.7%)
**Implant**			
Original implant salvaged	9 (75%)	9 (100%)	0 (0%)
Implant removed	2 (16.6%)	0 (0%)	2 (66.7%)
Substitution	1 (8.3%)	0 (0%)	1 (33.3%)
**Capsule Procedure**			
None	6 (50%)	6 (66.7%)	0 (0%)
Capsulectomy	6 (50%)	3 (33.3%)	3 (100%)
**Adjuvant Treatment**			
Chemotherapy	5 (41.6%)	4 (44.4%)	1(33.3%)
Radiotherapy	6 (50%)	5 (55.6%)	1(33.3%)
Hormonal Therapy	6 (50%)	5 (55.6%)	1 (33.3%)
Immunotherapy	2 (16.6%)	2 (22.2%)	0 (0%)
**Same QQ as Primary Tumor**			
Yes	6 (50%)	4 (44.4%)	2 (66.7%)
No	6 (50%)	5 (55.6%)	1 (33.3%)

## Data Availability

The data presented in this study are available on request from the corresponding author.

## Abbreviations

The following abbreviations are used in this manuscript:

IBBR	Implant-based breast reconstruction
IBCR	Ipsilateral breast cancer recurrence
WLE	Wide local excision
RM	Radical mastectomy
NSM	Nipple-sparing mastectomy
SSM	Skin-sparing mastectomy
DFS	Disease-free survival
OS	Overall survival
